# High Monocyte Count and Expression of *S100A9* and *S100A12* in Peripheral Blood Mononuclear Cells Are Associated with Poor Outcome in Patients with Metastatic Prostate Cancer

**DOI:** 10.3390/cancers13102424

**Published:** 2021-05-17

**Authors:** Anna-Maja Åberg, Sofia Halin Bergström, Elin Thysell, Lee-Ann Tjon-Kon-Fat, Jonas A. Nilsson, Anders Widmark, Camilla Thellenberg-Karlsson, Anders Bergh, Pernilla Wikström, Marie Lundholm

**Affiliations:** 1 Department of Medical Biosciences, Pathology, Umeå University, 901 85 Umeå, Sweden; annamaja@savar.se (A.-M.Å.); sofia.halin@umu.se (S.H.B.); elin.thysell@umu.se (E.T.); anders.bergh@umu.se (A.B.); pernilla.wikstrom@umu.se (P.W.); 2 Department of Radiation Sciences, Oncology, Umeå University, 901 85 Umeå, Sweden; ltjon@hotmail.com (L.-A.T.-K.-F.); jonas.a.nilsson@umu.se (J.A.N.); anders.widmark@umu.se (A.W.); camilla.thellenberg@umu.se (C.T.-K.)

**Keywords:** prostate cancer, metastases, monocytes, S100A9, S100A12, peripheral blood mononuclear cells

## Abstract

**Simple Summary:**

Prostate tumors are heterogeneous with unpredictable outcomes, ranging from harmless to aggressive, metastatic and deadly disease. Current diagnostic methods have limited ability to predict disease aggressiveness. New biomarkers are urgently needed to improve risk stratification, preferably involving non-invasive procedures. The aim of this prospective study was to assess the prognostic value of the inflammation markers S100A9 and S100A12 together with the monocyte count in blood samples from a cohort of 121 prostate cancer patients. We show that high monocyte count and high mRNA levels of *S100A9*, *S100A12* are associated with poor outcome in patients with metastases at diagnosis. Our results suggest that analysis of these factors in blood could identify patients who are in direct need of additional treatment to conventional androgen deprivation therapy (ADT).

**Abstract:**

Increasing evidence indicates calcium-binding S100 protein involvement in inflammation and tumor progression. In this prospective study, we evaluated the mRNA levels of two members of this family, *S100A9* and *S100A12*, in peripheral blood mononuclear cells (PBMCs) in a cohort of 121 prostate cancer patients using RT-PCR. Furthermore, monocyte count was determined by flow cytometry. By stratifying patients into different risk groups, according to TNM stage, Gleason score and PSA concentration at diagnosis, expression of *S100A9* and *S100A12* was found to be significantly higher in patients with metastases compared to patients without clinically detectable metastases. In line with this, we observed that the protein levels of S100A9 and S100A12 in plasma were higher in patients with advanced disease. Importantly, in patients with metastases at diagnosis, high monocyte count and high levels of *S100A9* and *S100A12* were significantly associated with short progression free survival (PFS) after androgen deprivation therapy (ADT). High monocyte count and *S100A9* levels were also associated with short cancer-specific survival, with monocyte count providing independent prognostic information. These findings indicate that circulating levels of monocytes, as well as *S100A9* and *S100A12*, could be biomarkers for metastatic prostate cancer associated with particularly poor prognosis.

## 1. Introduction

Prostate tumors are heterogeneous and with an unpredictable outcome, ranging from tumors causing no symptoms to aggressive, metastatic and deadly disease. Today, serum levels of prostate specific antigen (PSA), Gleason score of prostate cancer biopsies, and TNM stage are all used for prostate cancer diagnosis and prognosis [1,2]. However, in many patients these methods have limited ability to predict disease aggressiveness. New biomarkers are therefore urgently needed to improve risk stratification, preferably involving non-invasive procedures.

Several studies show presence of inflammatory cells in prostate cancer and suggest that macrophages potentiate prostate cancer progression [3,4,5,6]. In addition, high levels of macrophages in prostate biopsies are associated with prostate cancer progression after hormonal therapy [7]. Previous studies have also shown that systemic inflammation, measured by combining C-reactive protein and albumin, is associated with excess risk of death from prostate cancer [8]. This suggests that inflammatory cells and inflammatory markers in the circulation could have prognostic value.

S100A9 and S100A12 are calcium binding proteins that belong to a subgroup of S100 proteins called calgranulins or myeloid related protein, having several extra- and intracellular functions [9,10]. S100A9 and S100A12 are mainly expressed by neutrophils, monocytes, and activated macrophages and are thus linked to innate immune functions and inflammation [9,11,12,13]. S100A12 mainly acts as a functional homodimer in contrast to S100A9 that can be present as a homodimer or in a complex with S100A8 [11,13]. S100A9 and S100A12 mediate their effect by interacting with cell surface molecules such as Toll-like receptor 4 (TLR4) and receptor for advanced glycation end-product (RAGE) [14,15,16].

In addition to inflammatory cells, S100 proteins have also been detected in tumor cells in many types of cancers, such as breast, lung, bladder, kidney, thyroid, gastric, colorectal and oral, suggesting a possible role in inflammation-associated carcinogenesis [11,17].

There is sparse information regarding S100A12 and cancer, with no studies on S100A12 and prostate cancer to our knowledge. However, high stromal expression of S100A12 in hepatocellular cancer is associated with poor patient outcome [18], and a recent study shows that S100A12 expression in tissues of glioma patients is correlated to tumor grade and size [19]. Upregulation of S100A9, on the other hand, seems associated with tumor progression and metastasis in most cancer forms [11,17,20]. In line with this, we have previously shown that high density of S100A9 positive inflammatory cells in prostate cancer stroma is associated with poor outcome [21]. High S100A9 in prostate tumors is also associated with biochemical recurrence [22]. High protein and mRNA expression of S100A9 are also reported for prostate cancer tissue compared to surrounding benign tissue, and in high-grade compared to low-grade cancers [23].

In this study, we were interested to see if similar information could be gained from blood sample analysis. A previous study analyzing circulating S100A9 protein levels in serum showed higher levels of S100A9 in prostate cancer patients compared to healthy men [23]. Another study showed contradictive results and could not detect any difference in plasma levels of S100A9 between prostate cancer patients with different risk profiles and controls [24], thus confirming the need for further evaluation. Moreover, studies of mRNA expression signatures in whole blood from patients with castration resistant prostate cancer (CRPC) have indicated that a dysfunctional immune system might be a key factor in determining poor prognosis [25,26,27].

In this prospective study, the aim was to evaluate the level distribution of *S100A9* and *S100A12* in peripheral blood mononuclear cells (PBMCs) and its prognostic value in prostate cancer patients. As monocytes could be a source of S100 proteins in PBMCs, we also analyzed the prognostic value of the monocyte count by themselves and in relation to *S100A9* and *S100A12*.

Here we show that high expression of *S100A9* and *S100A12* and a high monocyte count are associated with poor outcome in patients with metastases at diagnosis.

## 2. Materials and Methods

### 2.1. Patient Samples

Within Uppsala-Umea Comprehensive Cancer Consortium (U-CAN) [28], patients diagnosed with prostate cancer were recruited and a written consent was signed. The study was approved by the Ethical Committee (Dnr 2013-57-31M), Umeå University, Sweden.

Blood samples were prospectively collected at time for diagnosis between the years 2013–2016, and before any treatment. In total, 121 patients representing different stages of the disease were included in the study. Two patients were excluded due to low RNA concentration, leaving a total of 119 patient samples for inclusion in the mRNA analysis together with 15 samples voluntarily donated from un-matched healthy men. Patients were defined to have low risk (LR), intermediate risk (IR), high risk (HR) or metastatic disease (M1) at diagnosis, according to Gleason score, TNM stage and serum PSA levels [29], as specified in Table 1. Bone scan was performed to evaluate M1 stage.

M1 patients (n = 30) were subjected to androgen deprivation therapy (ADT) and followed over time with time for PSA progression and death noted until last follow up in January 2020. PSA progression was determined according to the PCWG2 criteria [30] with PSA ≥ 2 ng/mL and a 25% increase from nadir. At first routine clinical follow-up within approximately 3 months (median: 98 days, quartiles: 70–124 days), new blood samples were collected in 24 cases.

### 2.2. Sample Preparation

Whole blood was collected in EDTA tubes and centrifuged within two hours as previously described [31]. The plasma was frozen in −80 degrees Celsius and saved for later analysis. Peripheral blood mononuclear cells (PBMCs) were isolated from the remaining blood sample by Lymphoprep density gradient centrifugation (Nycomed). The PBMCs were washed in PBS and 7 × 10^6^ cells were used for flow cytometry analyses (BD LSRII, BD Biosciences, Franklin Lakes, NJ, USA) and the remaining PBMCs were frozen in RNA-later (Qiagen) for RT-PCR analysis.

### 2.3. RNA Preparation

Isolation of total RNA from PBMCs was performed using RNeasy plus mini kit (Qiagen, Hilden, Germany) according to the manufacturer’s instructions and quantified with Nanodrop spectrophotometer (Thermo Scientific, Waltham, MA, USA). The RNA integrity was determined using Agilent 2100 BioAnalyzer (Agilent, Santa Clara, CA, USA).

### 2.4. qRT-PCR Analyses

The Superscript VILO cDNA Synthesis kit (Invitrogen, Carlsbad, CA, USA) was used for converting 420 ng total RNA to cDNA. In this initial study we analyzed the expression of *S100A9* (QT00018739) and *S100A12* (QT00005635) by using Quantitec Primer Assays (Qiagen, Hilden, Germany). The primers were evaluated with melting curves to ensure one PCR-product. RT-PCR reactions were performed on a TaqMan 7900HT (Applied Biosystems, Life Technologies, Carlsbad, CA, USA). Samples were analyzed in duplicate and coefficient of variance (CV) between the samples was calculated, with a mean of 3.51%. Relative gene expression was determined using the comparative Ct method, with *RPL13* (QT00067963) and *GAPDH* (QT00079247) (Qiagen) as standard reference genes, as described in the Applied Biosystems user bulletin [32]. On every plate a standard sample from the laboratory was used for interplate calibration.

### 2.5. Flow Cytometry Analysis

Flow cytometry was used to analyze CD14 positive monocytes. In total, 112 samples before treatment together with 15 samples from healthy men were analyzed. Extracted PBMCs were washed in FACS medium (PBS containing 3% bovine calf serum and 0.05% sodium azide) and incubated with Human TruStain FcX^TM^ (BioLegend, San Diego, CA, USA) for 10 min at room temperature to block Fc receptors. PBMCs were then stained with anti-CD14-fluorescein isothiocyanate (FITC) (clone M5E2, BD Biosciences) on ice for 30 min in round-bottom 96-well plates. Isotype-matched irrelevant Ab was used as a negative control. After washing in FACS medium, staining was determined by flow cytometry (BD LSRII; BD Biosciences). Data was analyzed with FACSDiva software (BD Biosciences) after gating on the monocyte and lymphocyte population in the FSC/SSC window, followed by elimination of the doublets based on FSC-W/FSC-H and SSC-W/SSC-H parameters and then examine CD14 positive cells in the FL1/FSC window. Data from 100,000 gated events was collected and are represented as the percentage of positive cells. To calculate monocytes per ml blood, percent CD14 positive cells were multiplied with total number of PBMCs per mL.

### 2.6. Plasma Analyses

Protein profiling of plasma samples from 96 patients (Table S1) and ten healthy controls had been previously performed by 384-plex antibody bead array analysis (Plasma Profiling Facility, SciLifeLab, Stockholm, Sweden) and panels of proximity extension assays (Olink Proteomics, Uppsala, Sweden). Here, data for S100A9 (HPA004193 on bead array) and S100A12 (EN-RAGE on Olink Inflammation panel) were retrieved and expressed as relative levels, while the comprehensive data will be presented elsewhere.

### 2.7. Statistical Analyses

Statistical analyses were performed using SPSS Statistics 27 (SPSS Inc., Chicago, IL, USA). The non-parametric Kruskal–Wallis *H* test was used for multiple comparisons. The non-parametric Mann–Whitney *U* test (continuous variables) and Fisher’s exact test (categorical variables) were used to compare differences between groups. Bivariate correlations were calculated using Spearman’s rank correlation test. The pairwise Wilcoxon rank sum test was used when comparing samples at diagnosis with samples 3 months after ADT treatment. Kaplan–Meier survival analysis was used to estimate cancer-specific survival and progression-free survival (PFS) after ADT treatment with the log-rank test for univariate comparison and Cox proportional hazard model for multivariate analysis. Survival was defined as the time in months between the date of diagnosis to the date of last follow-up (January 2020) or to the date of an event (PSA progression or prostate cancer specific death). Median *S100A9* (0.935) and *S100A12* (0.940) values were used as cutoff values to define high and low mRNA expression and median monocyte count (22.1 × 10^4^/mL blood) was used as cutoff to define high and low monocyte count. All statistical analyses were two-sided and *p*-values ≤ 0.05 were considered statistically significant.

## 3. Results

### 3.1. Prostate Cancer Patients with Metastases Show High Levels of S100A9 and S100A12

This prospective study included a total of 121 prostate cancer patients that were defined to have low risk (LR), intermediate risk (IR), high risk (HR) or metastatic (M1) disease, according to Gleason score, TNM stage and serum PSA levels (Table 1) [29].

**Figure 1:**
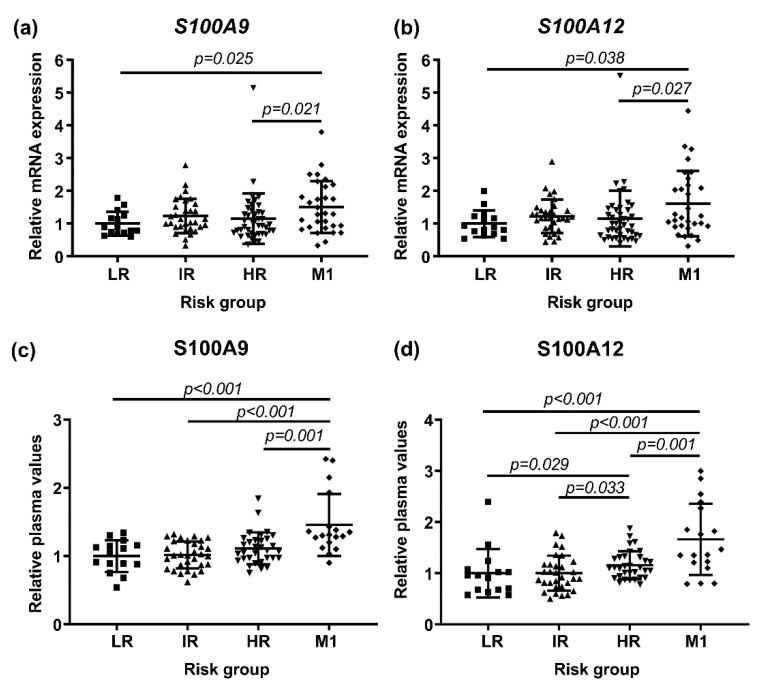
Evaluation of S100A9 and S100A12 according to patient risk group at diagnosis, stratified as low risk (LR), intermediate risk (IR), high risk (HR) and metastasis (M1). Shown are the relative mRNA levels of *S100A9* (**a**) and *S100A12* (**b**) in PBMCs; LR (*n* = 15), IR (*n* = 32), HR (*n* = 42), M1 (*n* = 30) and relative plasma protein levels of S100A9 (**c**) and S100A12 (**d**); LR (*n* = 15), IR (*n* = 31), HR (*n* = 32) and M1 (*n* = 18). Each dot represents one individual and horizontal bars indicate mean values with standard deviation. Group comparisons were performed using Kruskal–Wallis followed by the Mann–Whitney U test. Significant *p*-values from the Mann–Whitney U tests are indicated.

PBMCs were isolated from peripheral blood sampled at diagnosis of untreated patients and were characterized by RT-PCR for the expression of *S100A9* and *S100A12* in relation to patient risk group. Patients with diagnosed metastases had higher mRNA levels of *S100A9* and *S100A12* compared to the other risk groups (Figure 1a,b). Interestingly, these M1 patients had significantly higher expression of *S100A9* and *S100A12* compared to high risk patients without clinically detectable metastases (Figure 1a,b). Of note, *S100A9* and *S100A12* levels in LR, IR or HR patients were not significantly different compared to healthy men (*n* = 15) (Kruskal–Wallis; *p* = 0.063).

Plasma samples from a majority of the patients (Table S1) had been previously analyzed for broad protein profiling (unpublished), and data was now retrieved for S100A9 and S100A12. The plasma protein analyses showed similar results as the RNA analyses with significantly higher levels of S100A9 and S100A12 in patients with metastases compared to high risk patients without metastases (Figure 1c,d). The mRNA expression of *S100A9* and *S100A12* in PBMCs were correlated to each other and to their corresponding plasma protein levels (Table 2). However, the *S100A9* and *S100A12* levels were not correlated with age, Gleason score or PSA at diagnosis (Table 2).

The percentage of CD14 positive cells was analyzed by flow cytometry and calculated as monocytes per ml blood (monocyte count). The *S100A9* and *S100A12* mRNA levels were positively correlated with monocyte count, suggesting that monocytes could be a source of these factors (Table 2). No significant correlations were found between monocyte count and plasma protein levels of S100A9 (r = 0.125, *p* = 0.245) or S100A12 (r = 0.195, *p* = 0.067). Furthermore, no significant difference in monocyte count was found between the prostate cancer risk groups (LR, 14.3–34.0; IR, 10.6–31.0; HR, 10.3–25.8; M1, 12.1–34.0, 25–75 percentiles, Kruskal–Wallis; *p* = 0.658), although all prostate cancer patient risk groups had significantly higher monocyte count compared to healthy men (2.8–11.1, 25–75 percentiles, Mann–Whitney U; *p* < 0.001 compared to each respective group).

Taken together this suggests that S100A9 and S100A12 levels in blood could be markers of metastatic disease.

### 3.2. High Expression of S100A9 and S100A12 and a High Monocyte Count Are Associated with Poor Outcome in Patients with Metastases

We further examined *S100A9* and *S100A12* mRNA levels and monocyte count in relation to outcome in patients with metastases at diagnosis (n = 30). Outcome was analyzed based on two clinical endpoints; PSA progression after treatment with ADT or prostate cancer-specific death, with 22 patients having PSA progression and 11 patients dying from prostate cancer during the study period. Due to the low number of metastatic patients (n = 18, Table S1) we did not analyze the plasma protein levels of S100A9 and S100A12 in relation to outcome.

**Figure 2:**
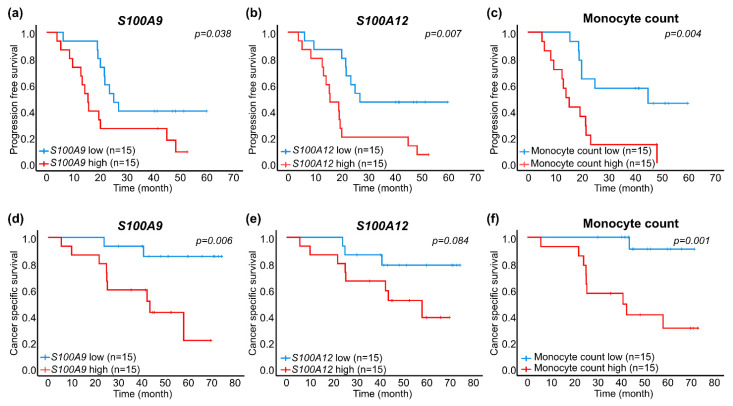
Kaplan–Meier survival analysis of patients in the metastatic group based on low (blue line) and high (red line) S100A mRNA expression and monocyte count, grouped by median values as cut off. Shown is PSA progression-free survival for *S100A9* (**a**), *S100A12* (**b**) and monocyte count (**c**), and prostate cancer-specific survival for *S100A9* (**d**), *S100A12* (**e**) and monocyte count (**f**).

Kaplan–Meier survival analysis with the log-rank test showed that patients with high mRNA expression (>median value) of *S100A9*, *S100A12* or a high monocyte count (>median value) had significantly shorter progression free survival than patients with low expression or low count (≤median value) (Figure 2a–c). Importantly, patients with high *S100A9* expression or a high monocyte count had significantly reduced cancer-specific survival compared to patients with low expression or low count (Figure 2d,f). A trend for reduced cancer-specific survival was also seen in patients of *S100A12* (Figure 2e).

In univariate Cox regression analysis, a high mRNA expression of *S100A12* was associated with increased relative risk for PSA progression (Table 3). *S100A9* and a high monocyte count were associated with increased relative risk for both PSA progression and prostate cancer-specific death (Table 3). Age, PSA at diagnosis or Gleason score were not significantly associated with progression free or cancer-specific survival in the M1 patients (Table 3).

In multivariate Cox regression analyses, only monocyte count remained significantly associated with increased risk of dying from prostate cancer in men with metastatic disease (Table 3). However, due to the small number of patients/events, all three factors could be important prognostic markers for metastatic prostate cancer and deserve to be further validated in larger patient’s cohorts.

**Figure 3:**
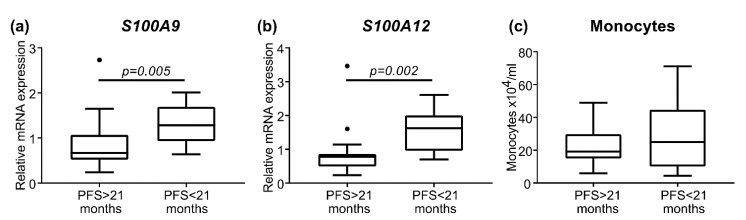
Short progression free survival (PFS < 21 months, *n* = 15) compared to long progression free survival (PFS > 21 months, *n* = 15) for patients with metastases at diagnosis. The relative gene expression levels of *S100A9* (**a**) and *S100A12* (**b**), and monocyte count (**c**) are illustrated by box plots. Outlier values (o) and significant *p*-values from the Mann–Whitney U tests are indicated.

The median time to PSA progression in the metastatic group was 21 months. Using 21 months as a cut off value, the patients were subdivided in short progression free survival (PFS < 21 months) and long progression free survival (PFS > 21 months), with 15 patients in each group (Table 4). There was a significant difference in *S100A9* and *S100A12* mRNA expression between the groups of short respective long progression free survival (Figure 3). In contrast, monocyte count was not significantly different between the groups (Figure 3). Moreover, the patients with long or short progression free survival differed in relation to overall survival, but not age, PSA, Gleason score or if patients had received early complementary chemotherapy (Table 4). This further indicates *S100A9* and *S100A12* mRNA expression as therapy-predictive factors for metastatic patients treated with ADT.

Taken together, this shows that high levels of *S100A9*, *S100A12* and high monocyte count in blood are associated with a particularly bad outcome in patients with prostate cancer metastases.

### 3.3. Expression of S100A9 Decreases after Treatment

To evaluate if ADT affected the mRNA expression levels of *S100A9* and *S100A12* or monocyte count, we analyzed blood samples collected from M1 patients (n = 24) approximately three months (median: 98 days, quartiles: 70–124 days) after treatment.

**Figure 4:**
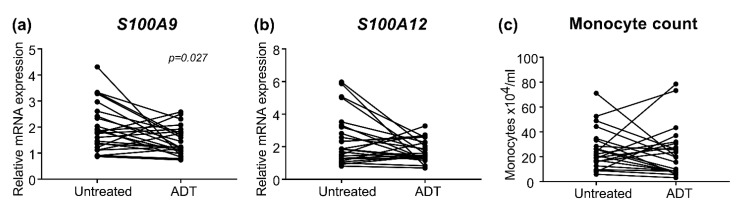
Evaluation in patients with metastases of (**a**) S100A9 and (**b**) S100A12 relative mRNA levels and (**c**) monocyte count in paired samples at diagnosis (untreated) and after approximately 3 months of androgen deprivation therapy (ADT). Wilcoxon rank sum test was used to compare samples from untreated metastatic patients at diagnosis with paired samples after ADT treatment. Significant *p*-values are shown.

Significantly reduced *S100A9* levels were seen in M1 patients treated with ADT (Figure 4a). However, the decrease in *S100A9* levels after ADT treatment was not correlated with PFS (r = −0.099, *p* = 0.646) or prostate cancer-specific death (r = −0.082, *p* = 0.704). Similar trends were seen for *S100A12* and monocyte levels, although not reaching significance (Figure 4b,c).

## 4. Discussion

In this prospective study, we have examined monocyte count and mRNA expression of *S100A9* and *S100A12* in PBMCs as well as corresponding circulating protein levels in blood samples from prostate cancer patients, and report higher levels in patients with metastases compared to high-risk patients without metastases. Importantly, in patients with metastases at diagnosis, high monocyte count and high mRNA levels of *S100A9* and *S100A12* are associated with short PSA progression-free time after ADT. Monocyte count and *S100A9* levels are also associated with short prostate cancer-specific survival, with monocyte count providing independent prognostic information. Furthermore, we show that metastatic patients that had early PSA progression (<21 months) had higher expression of both *S100A9* and *S100A12* compared to patients that progressed later (>21 months). These findings should be validated in a larger cohort of metastatic prostate cancer patients.

We show that mRNA expression of both *S100A9* and *S100A12* was positively correlated with monocyte count, suggesting that a population of monocytes could be one source of these proteins in PBMCs. In line with *S100A9* and *S100A12*, high blood monocyte count was shown to be associated with shorter PFS and cancer-specific survival in patients with metastases. Consistent with this, peripheral monocyte count has previously been shown as an independent prognostic biomarker for prostate cancer [33]. The absolute number of monocytes in peripheral blood has also been associated with survival in patients with B cell lymphoma [34] and locally advanced cervical cancer [35]. Furthermore, it was recently shown that monocyte subpopulation distribution and transcriptomes are significantly changed by the presence of endometrial or breast cancers and could reflect patient outcomes [36].

Taken together, our results suggest that analysis of monocytes or expression levels of *S100A9* or *S10012* in a blood sample could provide additive prognostic information for prostate cancer patients with metastases at diagnosis. In this group of patients, baseline PSA levels did not show any significant relation to patient outcome. Metastatic patients are in urgent need of novel treatments and high levels of *S100A9*, *S100A12* and monocytes in blood may not only identify patients in need of complementary therapies to ADT, but possibly also identify patients who would benefit from therapies affecting monocytes/macrophages and/or therapies targeting S100A9 or S10012 directly. Further studies to test these hypotheses are warranted.

The S100A9 inhibitor tasquinimod showed promising results in a phase 2 randomized controlled trial (n = 201), by improving both PFS and overall survival (OS) for metastatic CRPC (mCRPC) patients [37]. Inconsistent with these findings, a phase 3 randomized controlled trial (n = 1245) in men with chemotherapy-naïve mCRPC demonstrated that tasquinimod significantly improved radiographic PFS but did not improve OS [38]. In both these studies, tasquinimod was administrated to mCRPC patients blindly without selection of patients based on their S100A9 expression. Here we identified a group of patients with metastases that had high expression of S100A9, S100A12 and monocytes in blood, suggesting that this group of patients would likely respond better to tasquinimod or other treatments targeting this system. In relation to this, recent studies demonstrated that the frequency of peripheral blood monocytes in patients with melanoma, was associated with response to anti-CTLA-4 immune checkpoint blockade (ipilimumab) [39] and anti-PD-1 treatment [40].

We did not confirm if the levels of S100A9, S100A12 and monocytes in blood reflected an inflammatory profile in the prostate cancer tissue, but this is not unlikely. We have previously shown that S100A9 expression is detected in CD68 positive macrophages in the prostate tumor stroma and in the non-malignant prostate stroma adjacent to prostate tumors, suggesting monocytes/macrophages as a plausible source of S100A9 also in tumors [21]. Moreover, peripheral blood monocyte count has previously been correlated with the number of tumor-infiltrating macrophages [41]. However, prostate cancer cells and other cells, such as neutrophils and mast cells, have also been shown to express S100A9 [11,23,42,43]. Contribution of S100A9 and S100A12 from other cell types than PBMCs to the blood is therefore also likely and could explain the relatively low correlations between protein and mRNA levels for S100A9 and S100A12.

Moreover, we have previously shown that both S100A9 and CD68 positive macrophages are associated to poor outcome in PC patients [6,21]. We have also demonstrated that macrophages infiltrating PC are mainly of an M2 type (CD163^+^) and correlates with a more aggressive tumor and poor patient prognosis [3]. In experimental models, depletion of extratumoral macrophages/monocytes [44] and decreased S100A9 expression [45,46] inhibited prostate tumor growth. Taken together this shows the importance of macrophages in prostate tumor progression.

In this study, high monocyte count was associated with poor outcome in patients with metastases at diagnosis, suggesting that macrophages may be important for metastatic growth. We have previously shown that heme oxygenase-1 (HO-1)-positive macrophages are abundant in prostate bone metastases and at higher levels than in primary tumors [47]. Moreover, we have shown that infiltration of CD68 positive monocytes is higher in non-androgen receptor (AR)-driven CRPC bone metastases than in AR-driven, suggesting subgroups of metastases with a higher immune infiltration than others [48]. A recent study also demonstrated a progressive increase in mannose receptor-positive macrophages from normal prostate tissue to mCRPC [49]. Collectively, this indicates that monocyte count in blood could reflect the inflammatory profile in prostate cancer metastases.

Nevertheless, the inflammatory changes detected in blood in this study could also be due to systemic effects induced by metastatic disease that are unrelated to the levels within the tumors. More studies are needed to understand the mechanisms behind as well as the effects of the increased levels seen for circulating monocytes and expression of *S100A9* and *S100A12* in PBMCs in prostate cancer patients with particularly poor prognosis.

Furthermore, we show that *S100A9* expression in PBMCs was significantly decreased in M1 patients after ADT. The mechanism for this is unknown but indicate that ADT could directly or indirectly lower the inflammatory activity. However, no significant effect on the levels of *S100A12* or monocytes were found and the decrease in *S100A9* levels after ADT treatment was small and not correlated with outcome, hence not associated with response to treatment at the measured time-point. If *S100A9* expression change when prostate metastases become castration resistant needs to be further examined.

## 5. Conclusions

This is the first study showing that high blood monocyte count and high mRNA expression of *S100A9* or *S100A12* in PBMCs are associated with particularly poor outcome in patients with prostate cancer metastases, with monocytes providing independent prognostic information. Our results suggest that monocytes or the circulating levels of *S100A9* or *S10012* could be analyzed to give prognostic information for prostate cancer patients diagnosed with metastases, and possibly also to identify patients in need of complementary treatment to the conventional androgen-deprivation therapy. However, the study is limited by the low number of patients included, and further studies to test these hypotheses are warranted.

## Figures and Tables

**Table 1:** Clinical characteristics of prostate cancer patients.

Clinical Parameter	Low Risk (LR)	Intermediate Risk (IR)	High Risk (HR)	Metastasis (M1)
Number of patients	15	32	42	30
Age at first sample, median (quartiles) years	66 (59–69)	68 (62–72)	70 (67–73)	67 (63–79)
Initial PSA, median (quartiles) µg/L	6.2 (4.7–6.8)	5.3 (4.3–9.5)	28.5 (21–51)	193 (98–372)
Risk group *				
1: T 1–2 and GS < 7 and PSA < 10 µg/L	15			
2: T 1–2 and/or GS 7 and/or 10 ≤ PSA < 20 µg/L		32		
3a: T 1–2 and/or GS 8–10 and/or 20 ≤ PSA < 50 µg/L			17	
3b: T 3 and/or PSA < 50 µg/L			10	
4: T 4 and/or N1 and/or 50 ≤ PSA < 100 µg/L and M0			10	
4b: PSA ≥ 100 µg/L and M0			5	
5: Metastasis ^#^				30

* Risk groups were defined according to Gleason score (GS), TNM stage and serum prostate specific antigen (PSA) levels [29]. ^#^ Positive scintigraphy or bone scan at diagnosis.

**Table 2:** Bivariate correlations.

Variable	*S100A9* mRNA			*S100A12* mRNA		
	**r**		**n**	**r**		**n**
mRNA *S100A12*	0.879	**	119			
Gleason score	0.031		119	0.063		119
Initial PSA	0.101		119	0.055		119
Age	−0.097		119	−0.087		119
Plasma S100A9	0.307	**	94	0.322	**	94
Plasma S100A12	0.162		94	0.222	*	94
Monocyte count	0.239	*	112	0.258	**	112

Spearman’s rank correlation test. Data used in the correlation analyses was collected at the time of blood sampling before any treatment. Abbreviations: r, correlation coefficient; n, numbers; PSA, prostate specific antigen. * *p* < 0.05 (2-tailed). ** *p* < 0.01 (2-tailed).

**Table 3:** Cox regression analysis of specified markers in relation to PSA progression-free survival and prostate cancer-specific survival after androgen deprivation therapy of patients with metastases.

	PSA Progression			PC Specific Death		
Variable	HR	95% CI	*p*-Value	HR	95% CI	*p*-Value
(A) Univariate analyses						
*S100A9* mRNA	2.4	1.0–5.7	0.045	6.7	1.4–32	0.016
*S100A12* mRNA	3.2	1.3–7.9	0.010	3.1	0.8–12	0.100
Monocyte count	3.6	1.4–9.2	0.007	14	1.8–110	0.013
Age	1.0	1.0–1.1	0.186	1.1	1.0–1.1	0.059
Gleason Score	1.2	0.7–2.0	0.548	1.5	0.7–3.2	0.293
PSA	1.0	1.0–1.0	0.537	1.0	1.0–1.0	0.438
(B) Multivariate analysis						
*S100A9* mRNA	2.2	0.9–5.4	0.103	3.8	0.8–18	0.099
Monocyte count	3.4	1.3–8.7	0.011	10	1.2–82	0.031
(C) Multivariate analysis						
*S100A12* mRNA	2.9	1.1–7.6	0.027			
Monocyte count	3.3	1.3–8.5	0.012			

The *S100A9*, *S100A12* mRNA levels and monocyte count were dichotomized by their median values and analyzed as categorical variables. Age, Gleason score and PSA were analyzed as continuous variables. Abbreviations: HR, hazard ratio; CI, confidence interval; PC, prostate cancer; PSA, prostate specific antigen.

**Table 4:** Detailed clinical characteristics of metastatic prostate cancer patients treated with androgen deprivation therapy.

Clinical Parameter	Long PFS	Short PFS	*p*-Value
Number of patients	15	15	
PSA progression at last follow-up, *n*	7/15 (47%)	15/15 (100%)	
Age, median (quartiles) years	67 (65–76)	66 (61–80)	0.678 *
PSA at baseline, median (quartiles) µg/L	233 (91–330)	144 (115–391)	0.917 *
Gleason Score (Min-Max)	7–10	7–10	0.466 ^#^
Median time to PSA progression, (quartiles) months	42 (25–49)	14 (8.7–19)	<0.0001 *
Median time to death, (quartiles) months	53 (42–71)	41 (25–45)	0.011 *
Docetaxel prior to PSA progression, *n*	3	2	1.000 ^#^
Treatment post PSA progression			
Docetaxel, *n*	2	3	
Abiraterone, *n*	2	2	
Bicalutamide or Enzalutamide, *n*	3	7	
Radium-223, *n*		1	
No treatment, *n*		2	

Abbreviations: PFS, PSA progression-free survival after androgen deprivation therapy; *n*, numbers. Data are divided according to median time to PSA progression, long PFS > 21 months and short PFS < 21 months. * *p*-value obtained using Mann–Whitney test. ^#^ *p*-value obtained using Fisher test.

## Data Availability

Data are not publically availble due to Swedish law, GDPR restrictions for clinical data.

## Supplementary Materials

The following are available online at https://www.mdpi.com/article/10.3390/cancers13102424/s1, Table S1: Clinical characteristics of prostate cancer patients analyzed for plasma protein levels of S100A9 and S100A12.
