# Supplementary material for: High Monocyte Count and Expression of S100A9 and S100A12 in Peripheral Blood Mononuclear Cells Are Associated with Poor Outcome in Patients with Metastatic Prostate Cancer
Source: Cancers (Basel). 2021 May 17;13(10):2424. doi: 10.3390/cancers13102424 (PMC8156049; doi:10.3390/cancers13102424)
Supplement: Supplementary file 1 [file cancers-13-02424-s001.zip › cancers-1210446-supplementary.pdf]

Table S1. Clinical characteristics of prostate cancer patients analyzed for plasma protein levels of S100A9 and S100A12.

**Clinical parameter**	**Low risk (LR)**	**Intermediate risk (IR)**	**High risk (HR)**	**Metastasis (M1)**
Number of patients	15	31	32	18
Age at first sample, median (quartiles) years	66 (59-69)	67 (62-71)	70 (65-74)	67 (63-76)
Initial PSA, median (quartiles) µg/L	6.2 (4.7-6.8)	5.4 (4.3-9.5)	28 (17-37)	144 (99-372)
Risk group*				
1: T 1-2 and GS < 7 and PSA < 10 µg/L	15			
2: T 1-2 and/or GS 7 and/or 10 ≤ PSA < 20 µg/L		31		
3a: T 1-2 and/or GS 8-10 and/or 20 ≤ PSA < 50 µg/L			14	
3b: T 3 and/or PSA < 50 µg/L			8	
4: T 4 and/or N1 and/or 50 ≤ PSA < 100 µg/L and M0			8	
4b: PSA ≥ 100 µg/L and M0			2	
5: : Metastasis#				18

* Risk groups were defined according to Gleason score (GS), TNM stage, and serum prostate specific antigen (PSA) levels [29]. # Positive scintigraphy or bone scan at diagnosis.
